# Aplastic Anemia and Good Syndrome in a Heavily Treated Stage IV Thymoma Patient: A Case Report and Review of the Literature

**DOI:** 10.1155/2019/1910923

**Published:** 2019-11-12

**Authors:** Sofia Chiatamone Ranieri, Stefania Trasarti, Maria Antonietta Arleo, Luisa Bizzoni, Livia Bonanni, Valeria Di Battista, Maria Assunta Limongiello, Maria Grazia Nardacci, Giuseppe Gentile, Robin Foà

**Affiliations:** ^1^Department of Translational and Precision Medicine, University of Rome “Sapienza”, Rome, Italy; ^2^Department of Molecular Medicine, University of Rome “Sapienza”, Rome, Italy; ^3^Department of Medicine, Center of Hemato-Oncology Research (C.R.E.O.), University of Perugia, Perugia, Italy

## Abstract

Thymoma is an uncommon slowly growing neoplasm. It usually presents with paraneoplastic syndromes including the immunodeficiency syndrome called Good syndrome and hematological disorders. Pure red cell aplasia is a well-recognized complication of thymoma, and aplastic anemia is very rare in association with GS. We report a case of GS in a heavily treated patient with stage IV thymoma associated with a pure red cell aplasia and an amegakaryocytic thrombocytopenia that evolved into an AA and provide an up-to-date review of the relevant literature. This is the first case of the association of GS and AA with the coexistence of a heavily treated stage IV thymoma. The fatal outcome was not related to the progression of the thymoma, but rather to the severe infectious complications. The combination of lymphopenia and hypogammaglobulinemia typical of GS, coupled to the neutropenia, caused by bone marrow failure, was the main predisposing factor for the unfavourable outcome.

## 1. Case Presentation

In October 2015, a 53-year-old man was admitted to our hospital due to an incidental finding of severe anemia. The patient had a long medical history of thymoma (B2/B3) started in 1981 with pleural and lymphatic metastatic repetitions and Masaoka stage IVb and received chemo-, immuno-, and radiotherapy following multiple recurrences. He underwent five surgical excisions and five traditional lines of chemotherapy associated with radiotherapy to the mediastinum and paravertebral tissue. Due to the progression of the disease, he was enrolled in different experimental protocols including receptor radionuclide therapy with somatostatin analogue, sorafenib, volasertib, nintedanib, and everolimus. From October 2013, he started a maintenance treatment with low-dose cyclophosphamide plus octeotride. Periodic CT/PET scans showed a stable disease with paracostal, paracardiac, paravertebral, and paramediastinic residual metastatic nodules.

**Figure 1:**
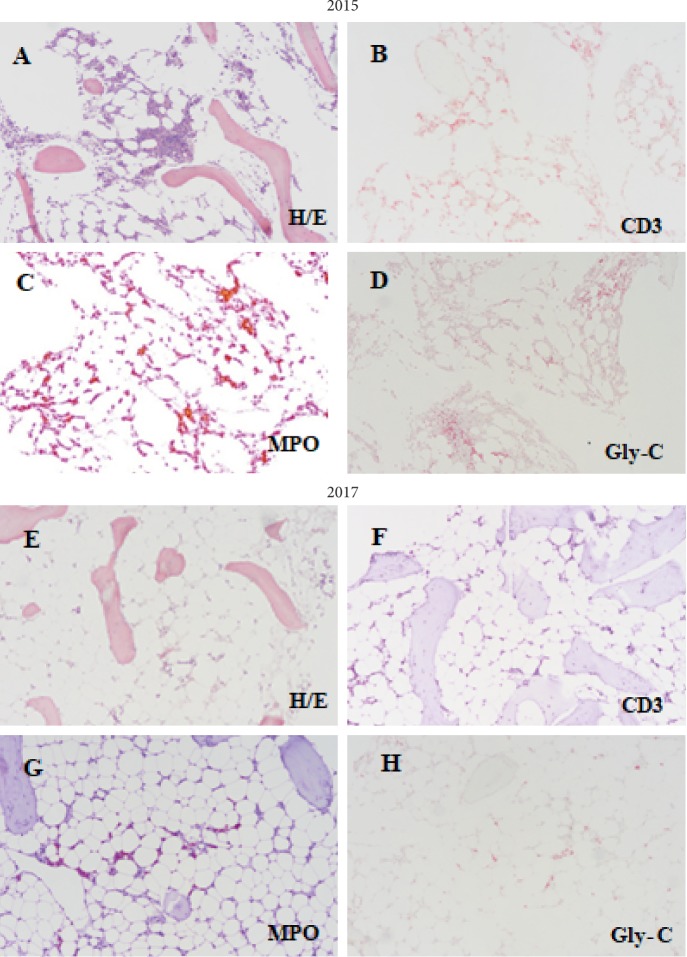
Histopathological images of the bone marrow (magnification 10x). (a) Biopsy section obtained on October 2015 showing a hypocellular bone marrow with preserved megakaryopoiesis (hematoxylin and eosin stain). (c) Myeloperoxidase staining revealed adequate numbers of myeloid cells. (d) Glycophorin C staining revealed a virtually absent erythropoiesis. (e) Biopsy section obtained on March 2017 revealed a severe hypocellular marrow (<5%) (hematoxylin and eosin stain). (g) Myeloperoxidase staining revealed a virtually absent myelopoiesis. (h) Glycophorin C staining revealed a virtually absent erythropoiesis. (b and f) In both biopsies, immunohistochemical stain for CD3 revealed a modest lymphoid component with interstitial distribution (CD3+).

When the patient was admitted to our institute (October 2015), the complete blood count was as follows: red blood cells 2.08 × 10^12^/L, hemoglobin 6.9 g/dL, mean corpuscular volume 95 fL, mean hemoglobin concentration 34.1 g/dL, white blood cells 6.7 × 10^9^/L (neutrophils 51% and lymphocytes 7%), platelets 447 × 10^9^/L, and reticulocyte count 0.2%. Serologies and DNA tests were negative for viral infections (CMV, EBV, HIV, and Parvovirus). No immunological abnormalities were detected, except for positive antiacetylcholine receptor antibodies and reduced levels of immunoglobulin isotypes IgG, IgA, and IgM. A bone marrow aspirate and biopsy revealed a virtually absent erythropoiesis with a preserved granulocytic and megakaryocytic maturation without infiltration by thymocytes and CD1a + lymphocytes (Figure 1-2015). A myelodysplastic syndrome was excluded by morphological evaluation, cytogenetic analysis, and mutational analysis, which revealed a normal karyotype and the absence of EZH2, GATA2, and TET2 mutations [1]. We also searched for rare mutations associated to sideroblastic anemia such as TRNT1. On this basis, we excluded the diagnosis of congenital sideroblastic anemia.

The study of the peripheral blood lymphocyte subpopulations showed the absence of B lymphocytes and an inversion of the CD4/CD8 ratio within the sCD3+ T lymphocytes, without a clonal restriction. Based on these findings, a diagnosis of PRCA associated to GS was made [2]. Cyclophosphamide was discontinued, and the patient remained on octeotride. The patient started supportive therapy with weekly blood transfusions, subcutaneous gammaglobulin (IG) infusions every three weeks, and erythropoietin and prednisone 75 mg daily. The last two drugs were suspended after 2 months due to lack of response. In May 2016, the patient developed a severe thrombocytopenia (platelets 15 × 10^9^/L). A bone marrow aspirate revealed a new marked reduction in megakaryocytes, and data were compatible with a diagnosis of AATP [3]. Treatment with high-dose ivIG (1gr/Kg) and steroids was started, which was followed by a slow and partial response that persisted for 9 months. Unfortunately, in February 2017, the patient showed again a severe thrombocytopenia (platelets 20 × 10^9^/L), unresponsive to steroids and high-dose ivIG. After Institutional Ethical Committee approval, eltrombopag was started as compassionate therapy at 50 mg daily dose and thereafter increased to 100 mg daily. After 4 weeks, the patient presented a marked neutropenia, and a bone marrow biopsy showed a final evolution to AA (Figure 1-2017).

Treatment with growth factors was initiated without benefit. The patient was not considered eligible to transplant because of the persistent thymoma and severe immunodepression due to GS and previous multiple chemotherapic treatment, so antithymocyte globulin was proposed [4]. After the prephase treatment with high-dose steroids, the patient developed a digestive bleeding associated to an invasive aspergillosis with fever and a severe hemorrhagic cystitis. The patient finally died of infectious complications.

## 2. Discussion and Conclusions

To our knowledge, this is the first case of the association of GS and AA with the coexistence of a heavily treated stage IV thymoma. GS is a rare association of thymoma and immunodeficiency first described more than 50 years ago. It is defined by low or absent B-cells in the peripheral blood, hypogammaglobulinemia and (in some cases) defects in cell-mediated immunity (CD4 + T lymphopenia and an inverted CD4 + /CD8 + T-cell ratio), and susceptibility to infections [2]. PRCA, thought to be due to T-cell-mediated destruction of erythroid progenitor cells, is reported in 2% of patients with thymoma and is characterized by anemia, reticulocytopenia, and severe bone marrow erythroid hypoplasia [2]. AATP is a rare immune-mediated disorder characterized by thrombocytopenia resulting from an unexplained reduction in the number of bone marrow megakaryocytes in the presence of an otherwise normal hematopoiesis. The exact prevalence of amegakaryocytic thrombocytopenia is unknown. The available literature comprises case reports and small case series [5]. AA is an uncommon complication of thymic tumors (0–1.4%). It is characterized by bone marrow failure. Treatment of PRCA, AATP, and AA is based on immunosuppressive drugs; there is, however, no consensus on the optimal therapeutic strategy [2, 4, 5]. The association of GS, PRCA, and AATP evolving into an AA is extremely rare. There is a series of cases reported with a concomitant PRCA and GS [6], cases of thymoma with AA [7], and 7 cases of PRCA and AATP [3, 8–13], 3 of which progressed to AA [3, 8, 9], but never with evidence of a GS.

Very few cases of AA in GS have been described in the literature with many of them being associated with benign spindle cell tumors [14–16], different from our case (Table 1). In 3/6 cases, the diagnosis of GS and AA was simultaneous with the diagnosis of thymoma [14, 15, 18], and in 1 case, thymectomy was performed but without remission of the AA [14]. In 2 cases, the diagnosis of GS associated with a PRCA which then evolved into an AA was made years after the onset of the thymoma [16, 17]; in one of the 2 cases, surgery and local chemotherapy instillations were carried out after the onset of anemia, but they were ineffective for aplasia although there was no recurrence of thymoma [17]; and in the other case, there are insufficient data to define the state of the illness [16]. Five of the 6 cases died of infectious and hemorrhagic or thrombotic complications. In the more recently described case of pancytopenia, this appeared three years after thymectomy and without evidence of cancer recurrence, and the patient responded to eltrombopag [19].

In a recent review of AA and thymoma, cyclosporine therapy, both either alone or in combination with other agents such as ATG and G-CSF, was associated with a response rate of 66.6% [7]. Furthermore, there are 4 reports which have described patients who underwent an allogenic hematopoietic stem cell transplantation for aplastic anemia, 2 of which with metastatic thymoma, nobody with GS. All showed a good response [8, 20–22]. Considering the refractoriness of the tumor, in our case the administration of immunosuppressive agents to prevent tumor progression was excluded [23]; unfortunately, the response to corticosteroids and high-dose iv IG was ineffective for the PRCA and partially effective for the AATP and failed to contain the progression to AA.

ATG was proposed, but the patient died after the prephase treatment due to invasive aspergillosis. In view of the promising results obtained in AA [24] and in a case of GS with pancytopenia [19], we attempted to use eltrombopag, but could not assess its effectiveness due to the short time of administration. The prognosis of GS is considered worse than that of other primary immunodeficiencies, with an overall mortality rate of 46% [2].

The combination of lymphopenia and hypogammaglobulinemia typical of GS, coupled to the neutropenia, caused by bone marrow failure, was the main predisposing factor for the poor outcome of our patient [25].

In the literature, there have been 30 cases of AATP without significant comorbidities and autoimmune disease that have also shown a rapid progression to AA [26]. The rapid progression of AATP to AA in these cases suggests that thymoma-associated AATP may have a more aggressive disease course than thymoma-associated PRCA. In our case, the previous and prolonged chemo-, radio-, and immunotreatment could have caused a severe immunodepression with an acquired immunodeficiency syndrome (as observed, for example, in patients transplanted or subjected to long-term immunosuppressive treatments) associated with clonal/dysplastic changes responsible for the aplasia as observed in our patient. For this reason, our first goal was to exclude a myelodysplastic syndrome secondary to chemotherapy and radiotherapy treatments: no morphological changes suggestive of MDS were observed, the karyotype was normal, and also the mutations associated with myelodysplasia and rare forms of sideroblastic anemia associated with immunodeficiency such as TET2, GATA2, TRNT1, were negative.

On the other hand, the presence in the bone marrow of an expansion of T lymphocytes in the absence of clonality, the inversion of the CD4/CD8 ratio observed at the time of immunophenotyping, and the selective absence of B lymphocytes associated with the presence of selective red series aplasia led us to believe that it was a paraneoplastic picture of altered maturation and disreactivity of autoimmune T lymphocytes. Before our clinical evaluation, we had no evidence of an immunoglobulin dosage, so we could not date the onset of Good syndrome. We only know that the patient has never had serious infections during previous treatments.

Every time a recurrence of the thymic disease occurred in our patient, therapy was immediately started (chemotherapy, radiotherapy, or immunotherapy) and complete remission was readily observed. Instead, at the time of our evaluation, the patient presented with a persistent although stable thymic-metastatic disease for at least 2 years, in maintenance with octeotride and cyclophosphamide. Therefore, it is assumed that the persistence of the thymic disease resulted in secondary pathologies such as PRCA, than evolved in AA and GS.

Our case highlights the challenging issue of treating a young fit patient with GS and AA, for whom, to date, we have no effective therapeutic tools. We describe this case not only for its rarity, but also for the complexity of the clinical management in an attempt to improve our understanding of this difficult-to-treat entity.

## Figures and Tables

**Table 1:** All reported cases of concomitant Good syndrome and aplastic anemia. The table is divided into seven horizontal rows illustrating reported cases including ours and twelve vertical columns illustrating literature, patient age and sex, disease progression, year of presentation of AA in relation to the diagnosis of thymoma, type of thymoma according to available WHO or traditional classification, bone marrow histology, information on serum immunoglobulins and lymphocyte subtypes, related manifestations, information on treatment received for thymoma, treatment for AA, and outcome and cause of death.

Report	Age (years) sex	Disease progression	Aplastic anemia and diagnosis of thymoma	Thymoma histology and stage	Bone marrow	Gammaglobulin and lymphocytes	Related features	Therapy for thymoma	Therapy for AA	Outcome	Cause of death
Chapin [16]	67 M	PRCA^1^, GS^2^, aplastic anemia	10 years after the onset of thymoma	Spindle cell thymoma	Aplasia with lymphoid infiltrate	Hypogamma	Na	None	Steroids	Death	Hemorrhage
Korn et al. [17]	75 M	GS^2^+ agenerative anemia, pancytopenia	7 years after the onset of thymoma	Mixed thymoma with plasmacytoid cells locally invasive	Aplasia + aggregates of mature lymphocytes	Hypogamma and severe impairment of cell immunity	Anti-AchR+^‡^	Surgery and nitrogen mustard instillation after the onset of anemia	Steroids and hormones	Death	Pulmonary embolus
Rogers et al. [14]	60 F	GS^2^+ aplastic anemia	Simultaneous	Spindle cell encapsulated thymoma	Marked hyopoplasia + few lymphocytes and plasma cells	Hypogamma	Ana+^†^	Surgery after the onset of anemia	Steroids and hormones	Death	Pneumonia and sepsis
Burrows and Carroll [15]	90 M	GS^2^+ aplastic anemia	Simultaneous	Spindle cell locally invasive	Marked hypoplasia + aggregates mature lymphocytes	Slightly hypogamma	None	None	Steroids	Death	Sepsis
Mir et al. [18]	68 M	GS^2^+ aplastic anemia	Simultaneous	Na	Hypoplasia	Hypogamma and lymphopenia	Na	None	Na	Death	Na
Kristiansen et al. [19]	61 M	GS^2^, pancytopenia	3 years after the onset of thymoma	Na	Hypoplasia + *T* lymphocytosis	Hypogamma CD4/CD8 inverted	None	Surgery before the onset of aplastic anemia	ATG^*α*^, steroids, CSA^*β*^, CTX^*γ*^, ivIG^*δ*^, G-CSF^*ε*^, eltrombopag	Response to eltrombopag + G-CSF#	
Our case	53 M	GS^2,^, PRCA^1^, AATP^3^, aplastic anemia	34 years after the onset of thymoma	Thymoma B2-B3 IV stage	Aplasia with modest lymphoid infiltrate	Hypogamma CD4/CD8 inverted absent B lymphocytes	Anti-AchR+^‡^	Surgery radiation and chemotherapy before the onset of aplastic anemia	Steroids, ivIG^*δ*^, G CSF^*ε*^, eltrombopag	Death	Sepsis

Na: not available; 1 PRCA: pure red cell aplasia; 2 GS: Good syndrome; 3 AATP: amegakaryocytic thrombocytopenia; ^†^Ana+: antinuclear antibodies positive; ^‡^AchR+: acetylcholine receptor *antibodies positive*; ^*α*^ATG: antithymocyte globulin; ^*β*^CSA: cyclosporine; ^γ^CTX: cyclophosphamide; ^*δ*^ivIG: intravenous gammaglobulin; ^*ε*^G-CSF: granulocyte colony-stimulating factor.

## Abbreviations

Na: Not available
^†^Ana+: Antinuclear antibodies positive
^‡^AchR+: Acetylcholine receptor antibodies positive
^*α*^ATG: Antithymocyte globulin
^*β*^CSA: Cyclosporine
^*γ*^CTX: Cyclophosphamide
^*δ*^ivIG: Intravenous gammaglobulin
^*ε*^G-CSF: Granulocyte colony-stimulating factor
AA: Aplastic anemia
GS: Good syndrome
AATP: Amegakaryocytic thrombocytopenia
PRCA: Pure red cell aplasia
CT/PET: Computed tomography/positron emission tomography
CMV: Cytomegalovirus
EBV: Epstein–barr virus
HIV: Human Immunodeficiency virus
ATG: Antithymocyte globulin.
